# Using a novel cognitive screening test to identify dementia risk amongst community‐dwelling cognitively unimpaired older adults from underserved populations.

**DOI:** 10.1002/alz.091680

**Published:** 2025-01-03

**Authors:** Mario A Parra‐Rodriguez, Alfredis Gonzalez‐Hernandez, Jasmin Bonilla‐Santos, Dorian Cala, Duvan Gomez, Eduardo A Forero

**Affiliations:** ^1^ University of Strathclyde, Glasgow United Kingdom; ^2^ Surcolombiana University, Neiva, Huila Colombia; ^3^ Cooperative University of Colombia, Neiva, Huila Colombia; ^4^ University of Amazonia, Florencia, Huila Colombia

## Abstract

**Background:**

Recent studies suggest that among cognitively unimpaired older adults, there are individuals who might present with subtle cognitive impairments suggesting the risk of Alzheimer’s disease (AD; Bos et al., 2018). The Visual Short‐Term Memory Binding Test (VSTMBT) identifies older adults who are accumulating AD pathology even though they are otherwise cognitively unimpaired (Parra et al., 2024). We investigated whether community dwellers from different socioeconomic groups above their 60s and displaying subtle VSTMB impairments also present neuropsychological profiles compatible with dementia risk.

**Methods:**

A sample of 235 community‐dwelling older adults were assessed with an extensive neuropsychological battery (Bonilla‐Santos et al., 2023) and a sensitive scale of functional abilities (T‐ADLQ; (Muñoz‐Neira et al., 2012). We divided the sample following recent recommendations drawn from the VSTMBT (Parra et al., 2024). We found that out of the total sample, 43 participants were classified as normal binders (performance on two VSTMBT above cut‐off), 99 were classified as suspicious binders (performance on at least one VSTMBT below cut‐off), and 93 were classified as weak binders (performance on both VSTMBT below cut‐off).

**Results:**

Group comparisons revealed that relative to normal and suspicious binders, weak binders have poorer performance on cognitive screening tools (MMSE, *F*(2,230) = 4.01, p = 0.019; ACE‐R *F*(2,230) = 3.4, p = 0.035) and functional scales (T‐ADLQ, *F*(2,230) = 4.53, p = 0.012). Such effects were unaccounted for by years of education, gender, socioeconomic status, comorbidities, or toxic habits. Age was a significant predictor with weak binders being older (*F*(2,234) = 6.31, p = 0.002). Assessment of subjective cognitive experiences (SCD) revealed no group effects (*F*(2,202) = 0.57, p = 0.566). Standard neuropsychological tests from the CERAD revealed marginal group effects for variables previously acknowledged as markers of AD.

**Conclusions:**

The VSTMB test identified AD risk profiles in community‐dwelling older adults from an underserved population. The fact that such a test proved insensitive to gender, education, socioeconomic status, comorbidities and toxic habits, suggests that this assessment tool can be reliably used to identify, in community settings, older adults who are at risk of dementia even if they have not yet complained about their cognitive abilities or sought medical help.

